# Treatment outcomes of proton or carbon ion therapy for skull base chordoma: a retrospective study

**DOI:** 10.1186/s13014-018-1173-0

**Published:** 2018-11-26

**Authors:** Masaru Takagi, Yusuke Demizu, Fumiko Nagano, Kazuki Terashima, Osamu Fujii, Dongcun Jin, Masayuki Mima, Yasue Niwa, Kuniaki Katsui, Masaki Suga, Tomohiro Yamashita, Takashi Akagi, Koh-ichi Sakata, Nobukazu Fuwa, Tomoaki Okimoto

**Affiliations:** 1 Proton Therapy Center, Sapporo Teishinkai Hospital, 3-1, East-1, North-33, Higashi-ku, Sapporo, Hokkaido 065-0033 Japan; 2 Department of Radiology, Hyogo Ion Beam Medical Center, Tatsuno, Hyogo Japan; 3 Department of Radiation Oncology, Hyogo Ion Beam Medical Center Kobe Proton Center, Kobe, Hyogo Japan; 4 Department of Radiation Oncology, Hakodate Goryoukaku Hospital, Hakodate, Hokkaido Japan; 5 Proton Therapy Center, Tsuyama Chuo Hospital, Tsuyama, Okayama, Japan; 6 Department of Radiology, Okayama University, Okayama, Okayama Japan; 7 Department of Radiation Physics, Hyogo Ion Beam Medical Center, Tatsuno, Hyogo Japan; 8 Department of Radiation Physics, Hyogo Ion Beam Medical Center Kobe Proton Center, Kobe, Hyogo Japan; 9 Department of Radiology, Sapporo Medical University, Sapporo, Hokkaido Japan

**Keywords:** Skull base chordoma, Proton therapy, Carbon ion therapy, Radiotherapy, Surgery, Local control, Late toxicity

## Abstract

**Background:**

The usefulness of particle therapy for skull base chordoma has not been established. The aim of this retrospective study was to analyse the treatment outcomes of proton therapy (PT) and carbon ion therapy (CIT) in patients with skull base chordoma at a single institution.

**Methods:**

All patients who underwent PT or CIT with curative intent between 2003 and 2014 at Hyogo Ion Beam Medical Center were included in this study. Twenty-four patients were enrolled. Eleven (46%) received PT and 13 (54%) received CIT. Overall survival (OS), progression-free survival (PFS) and local control (LC) were calculated using the Kaplan–Meier method. Late toxicities were evaluated according to the Common Terminology Criteria for Adverse Events version 4.0.

**Results:**

The median follow-up was 71.5 months (range, 14–175 months). The five-year LC, PFS and OS rates were 85, 81, and 86%, respectively. The LC (*P* = 0.048), PFS (*P* = 0.028) and OS (*P* = 0.012) were significantly improved in patients who had undergone surgery before particle therapy. No significant differences were observed in the LC rate and the incidence of grade 2 or higher late toxicities between patients who received PT and CIT.

**Conclusions:**

Both PT and CIT appear to be effective and safe treatments and show potential to become the standard treatments for skull base chordoma. To increase the local control, surgery before particle therapy is preferable.

## Background

Chordoma is a rare tumour of the bone that arises from embryonic remnants of the notochord [1]. Approximately 25–35% of tumours are located at the base of the skull [2]. Due to their low metastasis rate of these tumours, local control (LC) is the most important indicator of patient survival [3]. Surgery is the primary modality; however, the location and the nature of invasive growth make it extremely difficult to completely remove the tumour [4, 5]. Postoperative or definitive radiotherapy has been conducted to enhance LC [6, 7]. Several studies have reported that chordomas are resistant to radiotherapy and require doses of 60 Gy or more for LC [8]. This dose level cannot be safely delivered by conventional radiotherapy using X-ray (XRT) as it exceeds the tolerance of surrounding organs at risk (OARs), including the spinal cord, brainstem, and optic pathways [9, 10].

For several decades, particle therapy, such as proton therapy (PT) and carbon ion therapy (CIT), have been used for skull base chordomas [11–18]. The positive physical characteristics of particle therapy include a Bragg peak and reduced lateral scatter, which enable a more conformal dose distribution compared with that of XRT [8]. As a result, particle therapies are considered to be able to increase local control and reduce severe late toxicities. Until the 2000s, surgery and postoperative XRT were considered standard treatment [6, 7, 19]. Given the results of several retrospective studies, particle therapies after surgery have become standard treatment for skull base chordoma [11–16]. However, mainly due to the rareness of the disease, the number of patients treated in one hospital is limited.

The aim of this study was to evaluate treatment outcomes in patients with skull base chordomas who received PT or CIT at our centre and to investigate the clinical role of particle therapy for the disease to add further evidence.

## Methods

### Study design and patients

We conducted an Institutional Review Board-approved, retrospective analysis of patients with skull base chordoma who received definitive PT or CIT between April 2003 and May 2014 at Hyogo Ion Beam Medical Center. The inclusion criteria for the present study were as follows: 1) histologically confirmed skull base chordoma, 2) no previous radiotherapy, and 3) a duration of follow-up ≥24 months for survivors. Twenty-four patients were enrolled. All eligible patients provided written informed consent before treatment.

### Radiation therapy

Patients were immobilised in the supine position with an adequate head angle using a custom-made thermoplastic cast. The target volumes and organs at risk were delineated on computed tomography (CT) and magnetic resonance imaging (MRI) fusion images. The clinical target volume (CTV) that included regions of suspected microscopic spread was generated around the gross tumour volume (GTV) by expanding three-dimensional margins of 5 mm anatomically and then using manual corrections based on anatomic structures. The planning target volume (PTV) was defined as the CTV plus a setup margin of 5 mm for PT and 3 mm for CIT. Radiation treatments were planned on a CT-based three-dimensional treatment planning system (FOCUS-M [CMS [St. Louis, MO, USA and Mitsubishi Electric, Tokyo, Japan] until April 2008 and Xio-M [CMS and Mitsubishi Electric] from May 2008 to May 2014).

Due to availability constrictions at the time, from April 2003 to April 2005, either PT or CIT was available. After April 2005, both beams were available and radiotherapy treatments with PT and CIT were simultaneously planned for each patient. After comparing dose distributions and the dose volume histogram (DVH), a team of radiation oncologists selected the more appropriate treatment for each patient. When comparing the radiation treatment plans between PT and CIT, the same total dose and fractionations were established. Dose constraints of OARs were considered the most important factor in selecting which treatment plan should be admitted. If doses to the OARs were similar, then the plan that had the favourable minimum dose to the CTV was selected. Both proton and carbon ion beams were administered using a passive delivery system (aperture, compensator, and range shifter wheel).

Radiobiological experiments at Hyogo Ion Beam Medical Center showed that the relative biological effectiveness (RBE) values for PT and CIT were 1.1 and 2 to 3.7, respectively (depending on the depth in the spread-out Bragg peak) [20]. In particle beam therapies, doses are reported as Gy (RBE), which is defined as the physical doses multiplied by the RBE of the protons and carbon ions. The selection of the total dose and fractionation was based on the tumour volume and the distances between the tumour and OARs. For both PT and CIT, the maximum dose constraints of the optic nerve, chiasma, cochlea, spinal cord and brainstem were 47 Gy (RBE) or less in equivalent dose in 2 Gy calculated using the linear-quadratic model with α/β = 3 [21]. Although there were no dose constraints for the brain, the radiotherapy planning was designed to minimise the dose to the brain as much as possible.

### Follow-up evaluation

The follow-up period was calculated from the initial date of particle therapy. Patients were evaluated at 3-month intervals for 1 to 3 years after the start of therapy and at 6-month intervals thereafter. Regular follow-up studies included physical examinations, endoscopy, diagnostic imaging (e.g., CT and/or MRI), and blood tests. Acute reactions and late toxicities were evaluated according to the Common Terminology Criteria for Adverse Events version 4.0 [22].

### Outcomes and statistical analysis

Continuous variables are presented as medians with ranges and categorical variables are shown as frequencies with percentages. The LC, progression-free survival (PFS) and overall survival (OS) rates were assessed for all patients using the Kaplan–Meier method. Local recurrence was defined as confirmed radiographic or clinical disease progression/recurrence on CT or MRI. Disease progression was defined as clinical or radiographic evidence of local, regional, or distant recurrence/progression. Survival was identified as the confirmed date of death or last follow-up. All statistical analyses were performed using SPSS Statistics 22 software (IBM, Armonk, NY, USA).

The following covariates were analysed for the relationship to LC, PFS, and OS using the log-rank test: age, gender, tumour status (primary vs. postsurgical recurrence), proximity of the tumour to the brainstem, surgical intervention, ion type, GTV volume, and minimum dose of GTV (GTV Dmin). In this study, tumours in which the PTV overlapped the brainstem were defined as proximal to the brainstem. The GTV Dmin was standardised to 2 Gy per fraction using the linear-quadratic model with α/β = 2 [21, 23]. Late toxicity rates in patients treated with PT and CIT were compared using Fisher’s exact test. *P*-values less than 0.05 were considered statistically significant.

## Results

### Patients

**Fig. 1:**
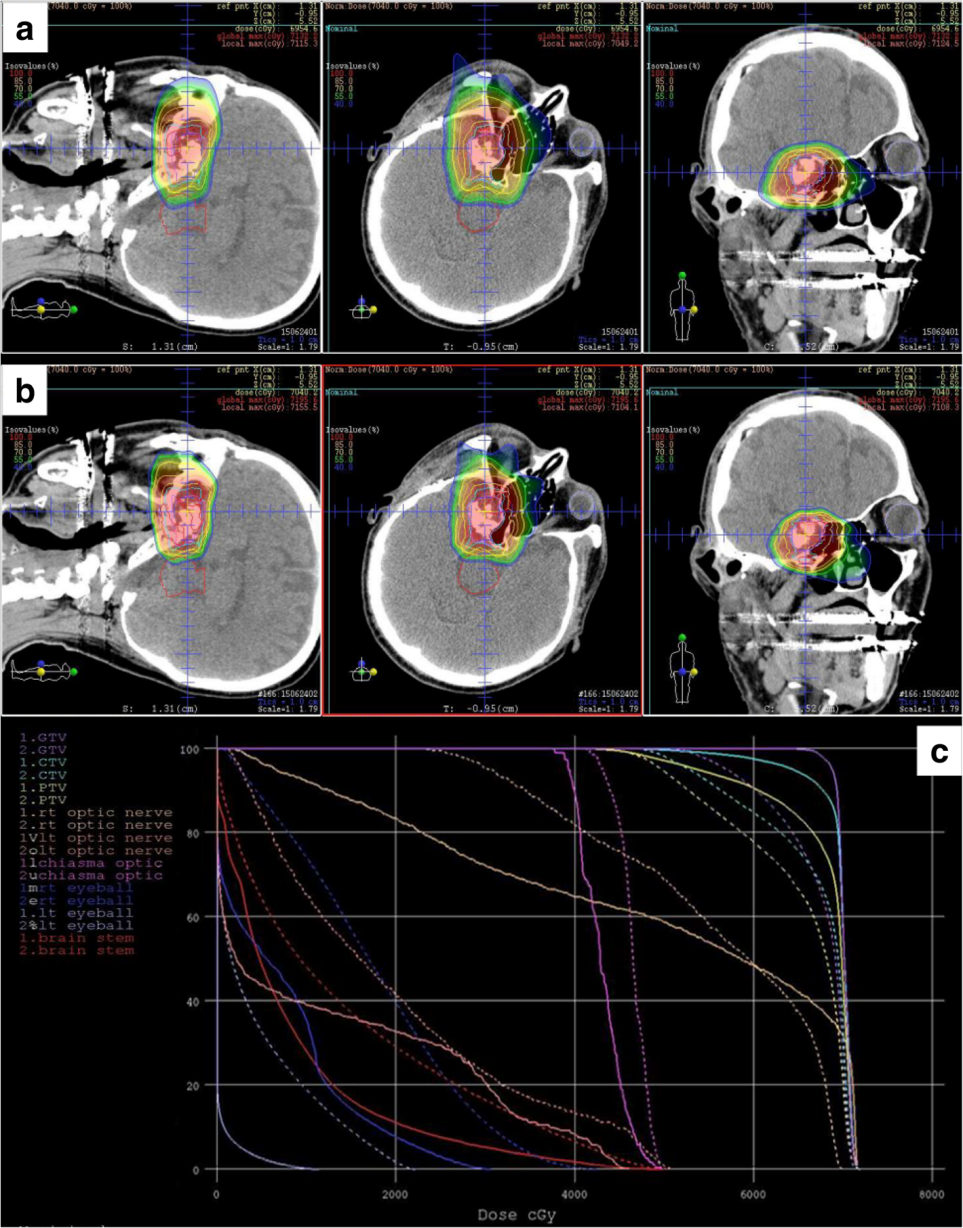
Comparison of proton (**a**) and carbon ion (**b**) treatment plans for the skull base chordomas. In the dose–volume histogram (DVH), the solid and dashed curves represent the carbon ion and proton plans, respectively (**c**)

Patient and treatment characteristics are shown in Table 1. The median follow-up period for all 24 patients was 71.5 months (range, 14–175 months). Before particle therapy, 14 patients (58%) received partial or subtotal resection, 10 patients (42%) received only biopsy and no patient received complete resection. Eleven patients (46%) received PT, and 13 patients (54%) received CIT. The median follow-up was longer in the PT group than in the CIT group (86 months vs. 56 months) without statistical significance (*P* = 0.902). In the PT group, 10 patients (91%) received 65.0 Gy (RBE) in 26 fractions, and patients in the CIT group received various doses and fractions. A representative dose distribution for a patient treated with 70.4 Gy (RBE) in 32 fractions is shown in Fig. 1. CIT was selected for this patient due to better coverage of the targets.

**Table 1:** Characteristics of Patients and Treatments

Characteristics		n	%
Total		24	
Age,	Median year (range)	55.5 (24–79)	
ECOG PS	0/1	21/3	88/12
Gender	Male/female	10/14	42/58
Tumour status	Primary/ postsurgical recurrence	17/7	71/29
Surgical intervention before PT or CIT	No/yes	10/14	42/58
Ion type	PT/CIT	11/13	46/54
GTV volume	Median ml (range)	17.0 (0.4–113.1)	
Total dose/ fractionation	57.6 Gy (RBE) / 16fr	1	4
	60.8 Gy (RBE) / 16fr	2	8
	65.0 Gy (RBE) / 26fr	13	54
	70.2 Gy (RBE) / 26fr	3	13
	70.4 Gy (RBE) / 32fr	3	13
	74.0 Gy (RBE) / 37fr	2	8
GTV Dmin^a^	Median Gy (RBE) (range)	55.0 (7.1–86.1)	

*PT* proton therapy, *CIT* carbon ion therapy, *GTV* gross target volume, *Dmin* minimum dose

^a^Minimum dose of GTV was standardised as equivalent dose in 2Gy with α/β = 2 using LQ model

### Survival and disease control

**Fig. 2:**
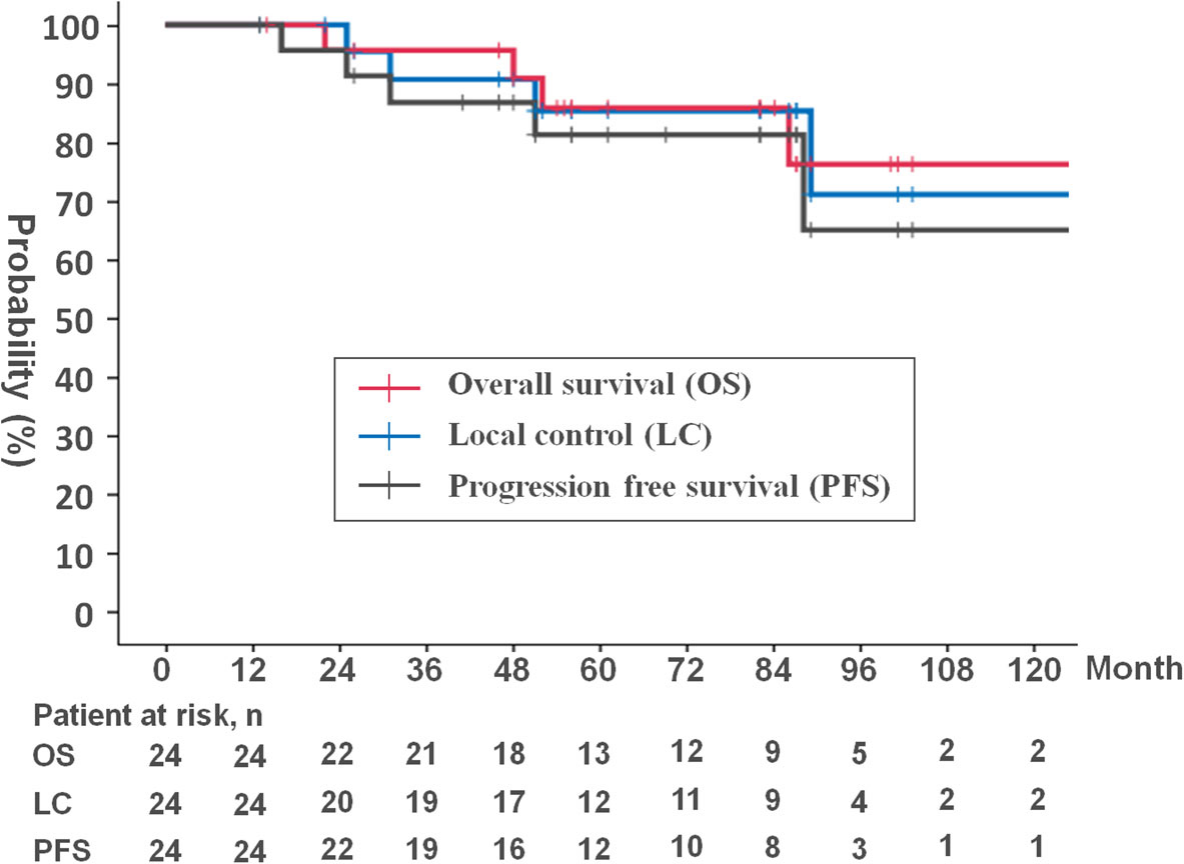
Kaplan-Meier analyses of local control (LC) (blue line), progression-free survival (PFS) (black line), and overall survival (OS) (red line) for all patients

Five patients (21%) experienced local recurrence with a median duration of 51 months (range, 25–155 months). All tumours recurred within their original GTV. Before particle therapy, all tumours were adjacent to the brainstem and the GTV Dmin values of these 5 patients were relatively low (median, 43 Gy [RBE]). After local recurrence, one patient received salvage surgery, and the disease was controlled at the final follow-up. One patient received re-irradiation using XRT after local recurrence; this tumour was not controlled. Three patients received no treatment after their local recurrence. The 5- and 8- year LC rates were 85% (95% confidence interval [CI]: 61–95%) and 71% (95% CI: 33–90%), respectively (Fig. 2).

Three patients (13%) experienced distant metastasis with a median duration of 69 months (range, 16–88 months): one with metastasis in the nasal cavity mucosa, one with bone metastasis and one with meningeal dissemination, respectively. The 5- and 8-year PFS rates were 81% (95% CI: 57–93%) and 65% (95% CI: 27–87%), respectively (Fig. 2).

At the last individual follow-up, 5 patients (21%) had died. Two patients died of the recurrent primary tumour and meningeal dissemination. Three patients died of intercurrent disease. The median time to death was 52 months (range, 22–175 months). The 5- and 8-year OS rates were 86% (95% CI: 62–95%) and 76% (95% CI: 46–91%), respectively (Fig. 2).

Based on the log-rank test, patients who underwent surgery before PT and CIT showed favourable LC (*P* = 0.048), PFS (*P* = 0.028) and OS (*P* = 0.012) (Table 2). There were no statistically significant differences observed in the LC (*P* = 0.752), PFS (*P* = 0.187) and OS (*P* = 0.060) between the PT and CIT groups (Table 2).

**Table 2:** Factors Prognostic of LC, OS and PFS

Factors		n (%)	LC		PFS		OS	
			5y (%)	*P*	5y (%)	*P*	5y (%)	*P*
Age	< 55	12 (50)	90	0.824	90	0.551	100	0.046
	> 55	12 (50)	81		73		74	
Gender	male	10 (42)	76	0.268	69	0.270	90	0.692
	female	14 (58)	92		92		83	
Untreated or recurrence	untreated	17 (71)	86	0.453	80	0.633	87	0.429
	recurrence	7 (29)	86		86		86	
Proximity of tumor and brainstem^a^	–	6 (25)	100	0.215	100	0.205	80	0.738
	+	18 (75)	81		75		81	
Surgery	–	10 (42)	57	0.048^*^	55	0.028^*^	68	0.012^*^
	+	14 (58)	100		100		100	
Ion type	PT	11 (46)	80	0.752	72	0.187	73	0.060
	CIT	13 (54)	92		92		100	
GTV volume	< 17.0 ml	12 (50)	91	0.776	91	0.482	91	0.059
	≥ 17.0 ml	12 (50)	77		70		82	
GTV Dmin^b^	< 55 Gy (RBE)	13 (54)	81	0.289	73	0.195	74	0.059
	≥ 55 Gy (RBE)	11 (46)	90		90		100	

*LC* local control, *PFS* progression-free survival, *OS* overall survival, *PT* proton therapy, *CIT* carbon ion therapy, *GTV* gross target volume, *Dmin* minimum dose

^*^Statistically significant

^a^Overlap of the PTV with the brainstem

^b^Minimum dose of GTV was standardised as equivalent dose in 2Gy with α/β = 2 using LQ model

### Acute reactions and late toxicities

Table 3 summarises acute reactions and late toxicities. All patients completed their PT or CIT without treatment delay caused by acute reactions. No patient experienced ≥ Grade 3 acute reactions.

**Table 3:** Acute reactions and late toxicities

	All (n)		
Acute reactions	Grade 1	Grade 2	
Dermatitis	8	3	
Mucositis	5	2	
Late toxicities	Grade 2	Grade 3	Grade 4
Brain necrosis		2	
Optic nerve disorder	3	1	
Nerve system disorders	1	2	
Hearing impaired	2		
Middle ear inflammation	2	1	
Pharyngeal hemorrhage			1

According to late toxicities, two patients experienced brain necrosis with grade 3 symptoms (mild cognitive and memory dysfunction). These symptoms improved with temporal oral corticosteroids. One patient whose tumour had infiltrated the optic canal experienced grade 3 unilateral blindness that had been predicted before particle therapy. One patient experienced grade 4 bleeding from an ulcer in the nasopharynx after CIT and was treated with coil embolisation.

Six patients in the PT group experienced ≥ Grade 2 (Grade 2:4, Grade 3:2) and 6 patients in the CIT group experienced ≥ Grade 2 (Grade 2:4, Grade 3:1, Grade 4:1) late toxicities. No statistically significant difference was observed with respect to ≥ Grade 2 late toxicities between the PT and CIT groups (*P* = 0.337).

## Discussion

To our knowledge, this is the first report describing the results of both PT and CIT treatments of skull base chordoma at a single institution. Both PT and CIT treatment showed favourable LC rates and tolerable late toxicities. Tumour control and survival were significantly improved in patients who had undergone surgery before particle therapy.

In this study, as the LC rate was favourable both in the PT and CIT groups with low rates of severe late toxicities, it is considered that both PT and CIT were effective and safe treatments. Currently, surgery followed by high-dose XRT is considered a standard treatment [24]. However, in most patients, XRT cannot deliver a sufficient dose to locally control the skull base chordomas due to the dose constraints of surrounding OARs. Based on recent guidelines for chordoma published in 2014 and 2015, it is recommended that at least 74 Gy (RBE) be delivered to the target volumes that are deemed to have microscopic disease and residual gross tumour after surgery [25, 26]. The physical characteristics of particle therapy, such as a Bragg peak and a sharper penumbra, can produce a more conformal dose distribution and making it possible to provide a higher dose of irradiation to the skull base chordoma without increasing the dose to adjacent OARs. Table 4 shows the results of other studies using XRT or particle therapy [6, 7, 11–16, 27]. In the guidelines developed by the European Sarcoma Network Working Group in 2014, due to the conformal dose distribution achieved by particle beam therapies, they should be considered the treatment of choice [25]. According to the position paper issued by the Chordoma Foundation, particle therapy is recommended instead of XRT due the better local control and survival [26]. In the guidelines presented in the position paper, XRT is considered acceptable only when dose uniformity in target volumes and doses to OARs similar to those with particle therapies can be achieved. Recently, Demizu et al. reported a 5-year LC of 73.8% in 53 skull base chordoma patients treated using PT in a retrospective multicentre study in Japan [28]. In this study, the LC rate was slightly improved and the incidence of late toxicities was similar to that in other studies using PT and CIT. Compared to XRT, the prescription doses were higher in particle therapies, and as a result, the local control rates were higher commensurately. The incidence of severe late toxicities was similar between XRT and particle therapies. Although these studies, including this study, were all retrospective, considering local control is the most important factor for patient survival, particle therapies, both PT and CIT, show potential to become standard treatments for skull base chordoma.

**Table 4:** Comparison of our findings with those of other studies

Author	Year	No.	F/U (m)	% of surgery^a^	Radiotherpay	Total dose	fractionations	Dose/fraction	5y-LC	5y-OS	Late complications
Debus [6]	2000	37	27	89	XRT	66.6	37	1.8	50	82	Hemiparasis: 1 patient
Zorlu [7]	2000	18	43	61	XRT	60	30	2	23	35	N.A.
Sahgal [27]	2015	24	36	93	XRT	76	38	2	65	86	Grade 3 hearing loss: 1 patient Grade 3 Hypopituitarism: 1 patient Radiation induced secondory malignancy: 1 patient
Hug [11]	1999	33	33	95	PT	64.8–79.2	36–44	1.8	59	79	Brain stem toxicity: 8% at 5 years Temporal lobe injury: 13% at 5 years Optic neuropathy: 4.4%
Ares [12]	2009	42	38	100	PT	67–74	N.A.	1.8–2.0	81	62	Grade 3 or 4 optic neuropathy: 2 patients Central nervous system necrosis: 2 patients
Hayashi [13]	2016	19	60	100	PT	77.44–78.4 ^b^	56–64	1.21–1.4	75	83	Temporal lobe necrosis: 1 patients
Schulz-Ertner [14]	2007	84	31	100	CIT	60–70	20	3.0–3.5	70	89	Grade 3 optic neuropathy: 4 patients Grade 3 necrosis of a fat plomb: 1 patient
Mizoe [15]	2009	19	33	N.A.	CIT	48–60.8	16	3.0–3.8	85	88	No patient experienced severe late toxicities.
Uhl [16]	2014	155	72	90	CIT	60	20	3	72	85	No patient experienced severe late toxicities.
This study	2018	11	86	36	PT	65.0–70.2	26	2.5–2.7	80	73	Grade 3 brain necrosis: 1 patient Grade 3 optic neuropathy: 1 patient
		13	56	77	CIT	57.6–74.0	16–37	2.0–3.6	92	100	Grade 3 brain necrosis: 1 patient Grade 4 bleeding: 1 patient

*LC* local control, *OS* overall survival, *XRT* photon therapy, *PT* proton therapy, *CIT* carbon ion therapy

^a^Including total, subtotal and partial resection. Only biopsy is excluded

^b^Using hyperfractionation

In this study, prognostic factor analysis showed better tumour control and survival in patients who underwent surgery before particle therapy. Other studies have also found that surgery prior to radiation therapy resulted in better outcomes [27, 29]. Hug et al. noted that surgery before PT made it possible to deliver a higher dose to the skull base chordoma even for patients with large tumours and disease-abutting crucial OARs [11]. Risks associated with surgery also exist. One patient who experienced nasal mucosal metastasis in this study was considered to show dissemination due to the surgical intervention. Some studies also reported patients who experienced dissemination in the nasal cavity after their surgery [11, 17, 30]. However, surgery before radiotherapy has two main advantages, reducing the tumour volume and separating the tumour from OARs. In this study, all tumours separated from the brainstem were locally controlled. Generally, surgery for the skull base chordoma often ends in incomplete resection. However, surgery before radiotherapy is considered eligible even with imperfect resection.

We found no statistically significant differences in the rates of local control and late toxicities between the PT and CIT groups, although the number of patients was small and their follow-up periods differed. As shown in Fig. 1, based on our experience, the CIT plans generally show a better dose distribution than PT plans due to their sharper penumbras. Moreover CIT is thought to have biological advantages over PT due to the higher RBE [20]. However, as carbon beams are irradiated from fixed ports, the beam angles of CIT were restricted. Clinically, in some patients, PT using gantry was more useful to avoid OARs. As shown in Table 3, there are no clear differences in the rates of local control and late toxicities between PT and CIT studies. However, all studies, including this study were retrospective, suggesting the need for additional prospective studies to explore the benefit of CIT.

Although the 5-year LC rate of 82% observed in this study was a favourable outcome, there is still room for improvement. It is considered indispensable to deliver a higher dose for local control of the skull base chordoma. Herman et al. summarized that the α/β value of the skull base chordoma was 2.0 and that local control rates were improved in proportion to the total dose [31]. Schulz-Ertner et al. reported that a dose of 75 Gy (RBE) or more that was standardised to 2 Gy per fraction using the linear-quadratic model with α/β = 2.0 would improve the local control rate in their CIT study results [14]. To deliver a higher dose, it is considered necessary to improve the radiation technique, such as dose distribution and radiotherapy schedule. Compared with the wobbler method, the spot-scanning method can create a more conformal dose distribution. Using the spot-scanning method, Ares et al. reported a 5-year LC of 81% in 42 skull base chordoma patients with low rates of late toxicities [12]. As the α/β values of the surrounding OARs were calculated to be 3.0, α/β values between chordoma itself and surrounding OARs are very close. It is considered that hypofractionation with an increased dose per fraction is not indicated. Hayashi et al. reported favourable LC in 19 patients who received hyperfractionation PT [13]. Further research is needed for an optimal dose and treatment schedule for skull base chordoma.

This study has several limitations. First, it was a retrospective analysis at a single institution. Second, chordoma is a disease that takes a very long time before recurrence, and the follow-up period in this study, although slightly longer compared with other studies, was not sufficient. Third, treatment heterogeneity was noted, including total dose and fractionation. Forth, due to the small number of patients, the impact of the statistical analyses, including the comparison between the PT and CIT groups, was relatively low. However, other published studies were also retrospective, and performing a prospective study is difficult due to the rarity of this disease. Therefore, we will increase the number of patients and continue to monitor these patients to report on follow-up data.

## Conclusions

The results of proton therapy and carbon ion therapy for skull base chordoma were both favourable regarding local control and late toxicities. Both showed the potential to become a standard therapy as opposed to XRT. To increase local control, surgery before particle therapy for tumour volume reduction and separation from OARs can be considered a viable alternative.

## Abbreviations

CI: Confidence Interval
CIT: Carbon Ion Therapy
CT: Computed tomography
CTV: Clinical Target Volume
Dmin: Minimum Dose
DVH: Dose Volume Histogram
GTV: Gross Tumor Volume
LC: Local Control
LQ model: Linear-Quadratic model
MRI: Magnetic Resonance Imaging
OAR: Organ at Risk
OS: Over All Survival
PFS: Progression Free Survival
PT: Proton Therapy
PTV: Planning Target Volume
RBE: Relative Biological Effectiveness
XRT: Conventional Radiotherapy Using X-ray
